# The synthetic lethal killing of RAD54B-deficient colorectal cancer cells by PARP1 inhibition is enhanced with SOD1 inhibition

**DOI:** 10.18632/oncotarget.13654

**Published:** 2016-11-26

**Authors:** Erin N. McAndrew, Chloe C. Lepage, Kirk J. McManus

**Affiliations:** ^1^ University of Manitoba, Department of Biochemistry & Medical Genetics, Winnipeg, Manitoba, Canada; ^2^ Research Institute in Oncology and Hematology, CancerCare Manitoba, Winnipeg, Manitoba, Canada

**Keywords:** cancer, RAD54B, PARP1, synthetic lethality, precision medicine

## Abstract

Colorectal cancer (CRC) is a leading cause of cancer-related death throughout the world. Despite improved screening efforts, most CRCs are diagnosed at late stages when surgery alone is not curative. Moreover, the low 5-year survival rate (∼8-13%) for those living with stage IV CRC highlights the need for better treatment options. Many current chemotherapeutic approaches are non-specific and associated with side effects due to their tendency to target both normal and cancer cells. To address this issue, synthetic lethal (SL) approaches are now being explored in cancer and are defined as the lethal combination of two independently viable mutations/deletions. From a therapeutic perspective, SL interactors of genes mutated in cancer serve as candidate drug targets. The present study focuses on *RAD54B*, a gene that is aberrantly expressed in many cancer types, including CRC. We show that *PARP1* silencing or inhibition (BMN673 or Olaparib) leads to selective killing within *RAD54B*-deficient cells relative to controls, and is accompanied by increases in γ-H2AX (a surrogate marker of DNA double strand breaks) and cleaved Caspase-3 (an apoptotic indicator). We further show that BMN673 synergizes with LCS-1 (an inhibitor of an established *RAD54B* SL interactor) to induce enhanced killing in *RAD54B*-deficient cells. Collectively, these data identify *RAD54B* and *PARP1* as SL interactors, and thus reveal PARP1 as a novel candidate drug target in *RAD54B*-deficient CRCs. These findings further show that combinatorial chemotherapies involving multiple SL targets may promote synergistic killing within cancer cells, a strategy that may hold potential in many cancer contexts.

## INTRODUCTION

Colorectal cancer (CRC) is the second leading cause of cancer-related deaths in North America. In 2015, it was estimated that ∼160,000 Americans and Canadians were newly diagnosed with CRC, with an additional ∼60,000 individuals ultimately succumbing to the disease [1, 2]. Based on these statistics it is evident that new therapeutic strategies are urgently needed to minimize the morbidity and mortality rates associated with the disease. Synthetic lethality is one such strategy and is defined as a rare and lethal combination of two independently viable mutations [3]. In a CRC context, a synthetic lethal (SL) approach aims to exploit a pre-existing gene deletion or mutation (e.g. *RAD54B*) in a cancer cell by down regulating a SL interactor (i.e. drug target). Since SL approaches are designed to exploit the aberrant genetics driving tumorigenesis, they are expected to better restrict their therapeutic effects to cancer cells than traditional approaches. Thus, identifying SL interactors of genes somatically altered in CRC will identify candidate drug targets with the potential to exploit and target cancer using a precision medicine strategy.

Recent gene re-sequencing efforts have identified numerous somatic mutations and deletions in genes encoding functions within the DNA damage response (DDR) [4] that are ideal targets to exploit via a SL paradigm. Disruption of the DDR correlates with genome instability [5], which accelerates the acquisition of subsequent mutations and/or gene copy number changes, and is a hallmark of virtually all cancer types [6, 7]. For example, *RAD54B* is somatically mutated or deleted in numerous cancer types, including CRC (∼3.3%) [8], breast (∼3.4%) [9], lung (∼2.6%) [10], which represents ∼20,500 North Americans each year who are newly diagnosed with these three cancers alone [1, 2]. *RAD54B* encodes a protein that functions in DDR, specifically within the DNA double strand break (DSB) repair pathway. In particular, RAD54B functions in homologous recombination repair (HRR) [11–14], which is commonly referred to as an ‘error-free’ repair pathway [11]. RAD54B is a member of the SWI/SNF2 helicase superfamily, and hydrolyses ATP to remodel protein-duplex DNA complexes to enhance the accessibility of chromatin to repair factors [15, 16]. RAD54B is also proposed to be an accessory factor for RAD51, that assists in HRR specifically during strand invasion into the undamaged sister chromatid [13, 17, 18]. Beyond HRR, *RAD54B* is also a chromosome instability (CIN) gene, as diminished expression induces CIN, or aberrant chromosome numbers [19]. Collectively, these data suggest diminished *RAD54B* expression and/or function are pathogenic events in the development and progression of cancer [20]. Importantly, these data suggest RAD54B may harbor tumor suppressor-like properties [19] rendering it an attractive target to exploit via a SL approach.

Although the clinical applicability of SL approaches is still in its infancy, numerous research groups have begun to uncover SL interactors (i.e. drug targets) for a myriad of genes somatically altered in cancer [21–23]. In fact, three SL interactors for *RAD54B* have already been identified and include *Flap Endonuclease-1* (*FEN1*) [19], *Superoxide Dismutase 1* (*SOD1*) [24], and *DNA Ligase IV* (*LIG4*) [25]. Collectively, these SL interactions rely on the synergistic killing of *RAD54B*-deficient CRC cells following the silencing or inhibition of a SL interactor that functions within the DDR (e.g. *FEN1, SOD1, LIG4*). These observations suggest that additional members of the DDR family may also be SL with *RAD54B*, including poly (ADP-ribose) polymerase 1 (PARP1). In 2005, two seminal studies identified *PARP* as novel drug target and SL interactor of *BRCA1* and *BRCA2*, which are commonly mutated in breast and ovarian cancers [19, 20]. Since then, subsequent studies have uncovered a number of additional SL interactions involving *PARP1* [19, 24, 26]. In particular, two high-throughput screens demonstrated that *PARP1* was SL with a large number of DDR genes including *CDK12*, *DDB1*, *XAB2*, however *RAD54B* was never identified [27, 28]. Due to the involvement of RAD54B within the DDR, we predicted *PARP1* would also be SL with *RAD54B*.

In this study, we couple siRNA-based silencing and small molecule inhibitors with semi-quantitative imaging microscopy, real time cell analyses (RTCA), and biochemical assays, and show that *RAD54B* and *PARP1* are SL. We show that *RAD54B*-deficient CRC cells are preferentially killed following *PARP1* silencing and inhibition with BMN673 and Olaparib. More specifically, we demonstrate that BMN673 and Olaparib treatments induce increases in γ-H2AX (a surrogate marker for DNA DSBs) preferentially within *RAD54B*-deficient cells and induce cytotoxicity via apoptosis. To enhance the therapeutic effects observed following PARP inhibition, we explored combinatorial treatments involving BMN673 with either 5-fluorouracil (5-FU), an established non-specific chemotherapeutic, or, LCS-1, an inhibitor of SOD1, a previously established SL interactor of *RAD54B* [29]. Although the combination involving 5-FU showed little enhanced effect beyond simple additivity, the combination involving LCS-1 induced synergistic killing within *RAD54B*-deficient cells. Collectively, our data show that *RAD54B* and *PARP1* are SL, and add *RAD54B* to the growing list of genes that can be therapeutically exploited with PARP inhibitors. Finally, our data also show that combinatorial approaches involving multiple SL targets can provide synergistic killing within CRC cells, and further suggest this combinatorial strategy may hold potential in other cancer contexts.

## RESULTS

### *RAD54B* and *PARP1* are synthetic lethal interactors

**Figure 1:**
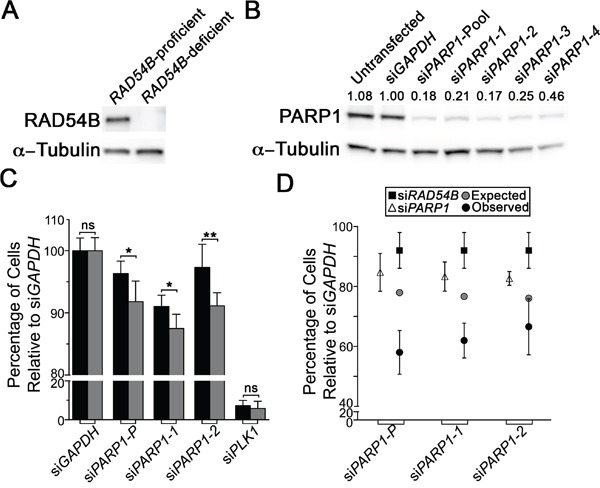
RAD54B and PARP1 are synthetic lethal interactors in HCT116 cells **A**. Western blot confirming *RAD54B* expression levels within the *RAD54B*-proficient and *RAD54B*-deficient cells; α-Tubulin serves as the loading control. Experiment was repeated two additional times. **B**. Western blot depicting diminished PARP1 expression in *RAD54B*-proficient HCT116 cells following silencing with either individual (si*PARP1-1*, *-2*, *-3* and *-4*) or pooled PARP1 (si*PARP1*-Pool) siRNAs or controls (Untransfected and si*GAPDH*); α-Tubulin serves as the loading control. Semi-quantitative analysis was performed and the normalized PARP1 expression levels relative to si*GAPDH* (1.00) are shown. Experiment was repeated twice. **C**. Bar graph depicting the mean normalized percentage of cells relative to si*GAPDH* (± SD) for *RAD54B*-proficient (black) and *RAD54B*-deficient (grey) cells following silencing with the indicated siRNAs (x-axis). Statistical significance is indicated (ns, not significant; *, *P*-value <0.05; **, *P*-value <0.01). Experiment was conducted in sextuplet and two additional times. **D**. Graphical depiction of the mean normalized percentage of cells relative to si*GAPDH* (± SD) for the individual silencing of either *RAD54B* (black squares) or *PARP1* (white triangles), and the expected combined value (grey circles) calculated using a multiplicative model. Black circles identify the observed values following the simultaneous silencing of *RAD54B* and *PARP1*, and reveal enhanced/synergistic effects beyond the expected values. Experiment was conducted in sextuplet and repeated two additional times.

Previous genetic studies have shown that a number of genes encoding functions within the DDR, particularly HRR, are SL with *PARP1* [27, 30–34]. Since a large number of genetic studies show that members of the same biological pathway frequently share SL interactors [19, 24, 26], we postulated *PARP1* would also be SL with *RAD54B*, as it also encodes functions within HRR [9, 35–37]. However, we first began by confirming *RAD54B* expression within the *RAD54B*-proficient (control) and *RAD54B*-deficient isogenic cell model (Figure 1A), and subsequently assessed four individual PARP1 siRNA duplexes to identify the two most efficient silencers, si*PARP1*-1 and si*PARP1*-2 (Figure 1B). Next, we queried whether PARP1 silencing induced preferential killing within the *RAD54B*-deficient cells relative to controls. The *RAD54B* isogenic model has been employed previously in similar siRNA-based SL studies [19, 24], and following silencing of a candidate interactor (e.g. PARP1) a decrease in the number of *RAD54B*-deficient cells relative to controls is suggestive of a SL interaction. Accordingly, cells were transfected with siRNAs, permitted to grow for ∼3.5 days, whereupon cells were fixed, imaged and analyzed. Following silencing, the relative percentage of *RAD54B*-deficient cells remaining was significantly reduced relative to controls (Figure 1C). In fact, the siRNA-Pool and both individual siRNAs induced statistically significant decreases in *RAD54B*-deficient cells (Table S1), suggesting *RAD54B* and *PARP1* are SL. Indeed, further scrutiny of the images revealed a subset of *RAD54B*-deficient cells exhibiting cytological hallmarks of cytotoxicity that are typical of apoptosis, including chromatin condensation and nuclear blebbing.

Although the above data imply *RAD54B* and *PARP1* are SL, it remains possible that the interaction results from a *de novo* background mutation that arose while generating the *RAD54B*-deficient cells. Since HCT116 cells are *MLH1*-deficient (i.e. mis-match repair defective) and mutations can accrue, the possibility remained that an additional mutation became clonally fixed within the *RAD54B*-deficient cells that accounts for putative *RAD54B PARP1* SL interaction. To alleviate this possibility, dual silencing experiments were performed in which both *RAD54B* and *PARP1* were either individually or simultaneously silenced within the parental *RAD54B*-proficient (control) cells. However, we first confirmed our ability to silence *RAD54B* [24] (Figure S1). Next, single (si*RAD54B* or si*PARP1*) and dual (si*RAD54B* plus si*PARP1*) siRNA experiments were performed and as predicted, the simultaneous silencing of *RAD54B* and *PARP1* induced a synergistic decrease in cell numbers compared to silencing either gene alone (Figure 1D), or that predicted using a multiplicative model (Table S2). Collectively, the above data show that *RAD54B* and *PARP1* are SL within HCT116 cells, and further identify PARP1 as a candidate drug target in a *RAD54B*-deficient CRC context.

### *RAD54B*-deficient HCT116 cells are hypersensitive to BMN673 and Olaparib

**Figure 2:**
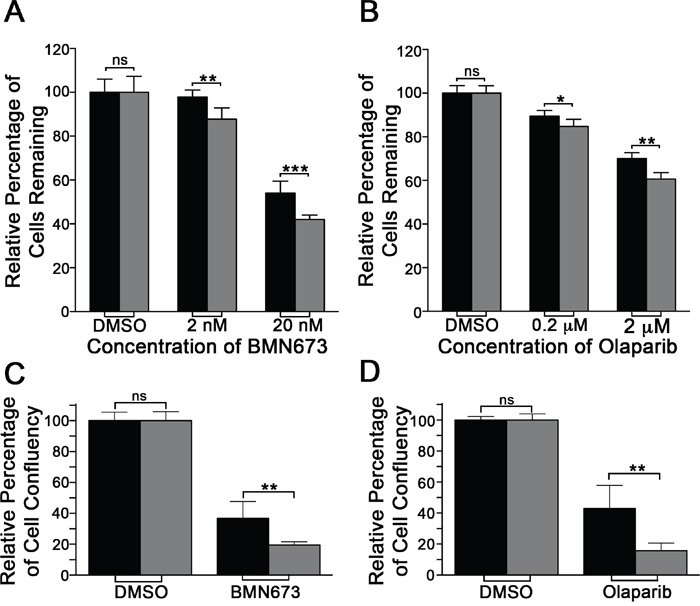
RAD54B-deficient cells are hypersensitive to BMN673 and Olaparib **A**. Bar graph depicting the mean (± SD) percentage of *RAD54B*-proficient (black bars) and *RAD54B*-deficient (grey bars) cells following DMSO or BMN673 treatment. Statistical significance is indicated (ns, not significant; **, *P*-value <0.01; ***, *P*-value <0.001). **B**. Bar graph presenting the mean (± SD) percentage of *RAD54B*-proficient (black) and *RAD54B*-deficient (grey) cells following DMSO or Olaparib treatment. Statistical significance is indicated (ns, not significant; **, *P*-value <0.01; ***, *P*-value <0.001). **C**. Bar graph depicting the mean (± SD) relative percentage of cell confluency for *RAD54B*-proficient (black) and *RAD54B*-deficient (grey) cells following DMSO (control) or BMN673 treatment. Data are presented normalized to the respective DMSO treated controls, and statistical significance is as indicated (ns, not significant; **, *P*-value <0.01). **D**. Bar graph of the mean (± SD) relative percentage of cell confluency for *RAD54B*-proficient (black) and *RAD54B*-deficient (grey) cells following DMSO (control) or Olaparib treatment. Data are presented normalized to the respective DMSO treated controls, and statistical significance is as indicated (ns, not significant; **, *P*-value <0.01). All experiments were conducted in sextuplet and repeated two additional times.

Next, we wished to determine whether two PARP inhibitors, BMN673 and Olaparib, could functionally substitute for *PARP1* silencing and induce preferential killing within the *RAD54B*-deficient cells. To begin, standard dose response curves were generated and revealed the *RAD54B*-deficient cells are hypersensitive to both inhibitors relative to controls. More specifically, the EC_50_ for BMN673 was 1.9-fold lower within the *RAD54B*-deficient cells (9.0 nM) than the control (17.5 nM), while for Olaparib it was 1.25-fold lower within the *RAD54B*-deficient cells (2.82 μM) relative to controls (3.53 μM). Subsequent Student's *t*-tests revealed statistically significant decreases in *RAD54B*-deficient cells following both BMN673 (Figure 2A; Table S3) and Olaparib (Figure 2B; Table S4) treatments. Thus, these data support those of the previous section, and further suggest BMN673 and Olaparib are chemogenetic (i.e. SL) interactors of *RAD54B*.

To confirm the above observations and validate BMN673 and Olaparib as chemogenetic interactors of *RAD54B*, mCFAs were performed. To best restrict the therapeutic effect to the *RAD54B*-deficient cells, the EC_50_ values calculated for BMN673 (9.0 nM) and Olaparib (2.8 μM) were employed in all subsequent work. Briefly, *RAD54B*-deficient and control cells were treated with compounds for 7 days and cellular confluency was quantitatively assessed. In agreement with the dose response curves, BMN673 and Olaparib treatments induced statistically significant decreases in confluency within the *RAD54B*-deficient cells relative to controls (Figure 2C and 2D). More specifically, BMN673 and Olaparib induced 1.9- and 2.7-fold decreases, respectively, within the *RAD54B*-deficient cells (Tables S5 & S6). These data confirm *RAD54B* and *PARP1* are SL and further identify BMN673 and Olaparib as lead therapeutic candidates warranting further pre-clinical investigation.

### BMN673 and Olaparib treatments induce proliferation defects in *RAD54B*-deficient cells

**Figure 3:**
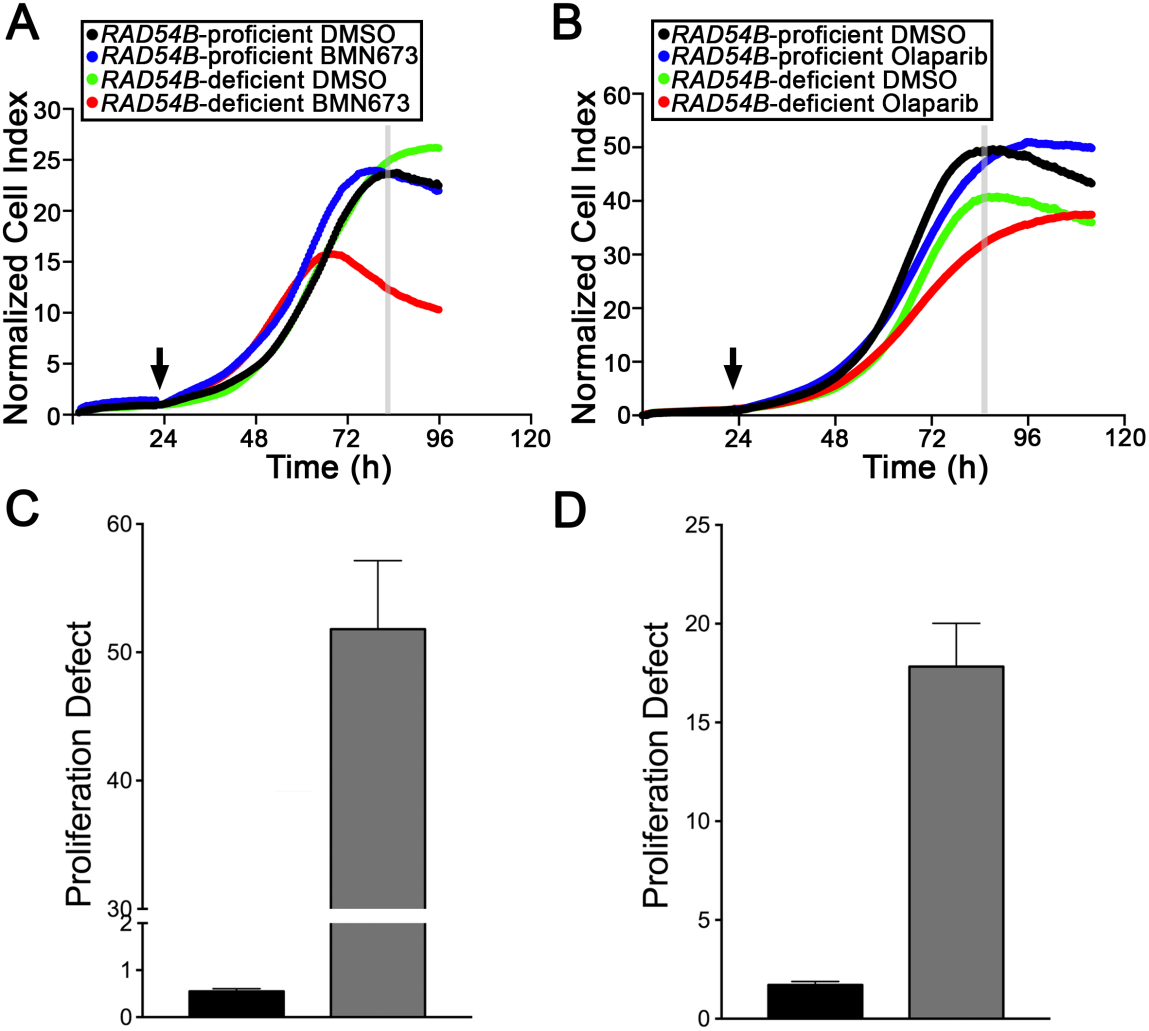
BMN673 and Olaparib treatments induce proliferation defects in RAD54B-deficient cells **A**. RTCA proliferation curves for control and *RAD54B*-deficient cells treated with either DMSO (vehicle control) or BMN673. The arrow identifies the time-point of compound addition, while the grey vertical line identifies the time-point employed to calculate the proliferation defect (∼84 h post-seeding). Experiments were performed in quadruplicate and repeated two additional times. **B**. RTCA proliferation curves for control and *RAD54B*-deficient cells treated with either DMSO (vehicle control) or Olaparib, with the arrow identifying the time-point of addition, and the grey vertical line identifying the time-point used to calculate the proliferation defect (∼88 h post-seeding). Experiments were performed in quadruplicate and repeated two additional times. **C**. Bar graph presenting the mean proliferation defect (± SD) calculated for the *RAD54B*-deficient (grey bars) cells treated with BMN673 relative to controls (black bars). **D**. Bar graph depicting the mean proliferation defect (± SD) observed within the *RAD54B*-deficient (grey) cells treated with Olaparib relative to controls (black).

Having established *RAD54B*-deficient cells are hypersensitive to both PARP inhibitors, we next sought to determine the underlying mechanism accounting for the decrease in cell numbers. Accordingly, RTCA was performed to distinguish whether cell cycle arrests (identified by a cell index plateau) or cellular cytotoxicity (identified as by either a change in slope [decrease] or rapid decline in cell index) were induced following treatments. RTCA employs electrical impedance as a measure of cellular proliferation and can easily discern cell cycle arrests (stationary phase identified by a plateau) from cell cytotoxicity [24, 38]. Figure 3 shows that the proliferation curves generated for the control cells are largely overlapping and virtually indistinguishable irrespective of treatment (DMSO, BMN673 or Olaparib). In contrast however, the proliferation curves are strikingly different within the *RAD54B*-deficient cells treated with BMN673 or Olaparib relative to vehicle control (DMSO), and are also suggestive of proliferation defects (PDs) rather than cell cycle arrests. For example, BMN673 treatment (Figure 3A) induces a rapid decline in cell index ∼40 h post-treatment within the *RAD54B*-deficient cells, while Olaparib treatment (Figure 3B) decreases proliferation as evidenced by a flattening of the slope during the exponential growth phase. The calculated PDs (Table S7) revealed a 94.2-fold and a 10.4-fold increase in mean PD within the *RAD54B*-deficient cells treated with BMN673 (Figure 3C) or Olaparib (Figure 3D), respectively. These data are in agreement with the previous section, and show BMN673 and Olaparib treatments induce PDs rather than cell cycle arrests.

### BMN673 and Olaparib treatments underlie increases in γ-H2AX and apoptosis in *RAD54B*-deficient cells

**Figure 4:**
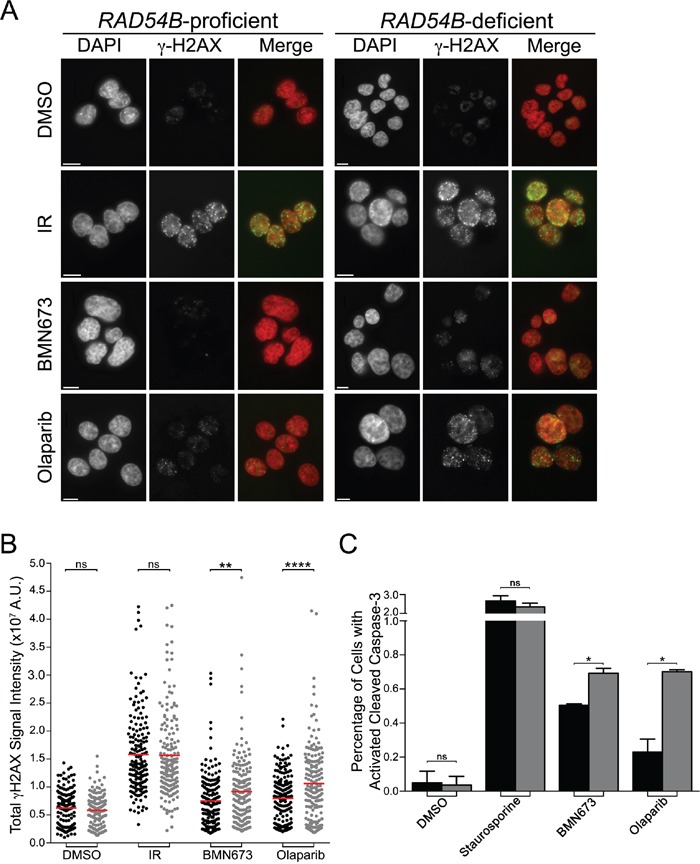
BMN673 and Olaparib induce preferential increases in γ-H2AX and cleaved Caspase-3 signal intensities in RAD54B-deficient cells **A**. Representative low resolution images depicting the abundance of γ-H2AX in cells treated with DMSO, IR, BMN673 or Olaparib. All images were acquired 48 h post treatments using the identical exposure times and thus changes in fluorescence intensities reflect changes in the global abundance of γ-H2AX (i.e. DNA DSBs). Nuclei and γ-H2AX are pseudocolored red and green, respectively, within the merge. Scale bar represents 10 μm. **B**. Scatter plots presenting the total γ-H2AX signal intensities from individual *RAD54B*-proficient (black circles) and *RAD54B*-deficient (grey circles) cells treated with DMSO (negative control), IR (positive control), BMN673 or Olaparib as determined by semi-quantitative IIF microscopy. A minimum of 175 cells were imaged per condition and the red bars identify the mean γ-H2AX signal intensity. Student's *t*-tests reveal statistically significant increases in γ-H2AX signal intensities in the *RAD54B*-deficient cells treated with BMN673 and Olaparib relative to corresponding *RAD54B*-proficient controls. (ns, not significant; **, *P*-value <0.01; ****, *P*-value <0.0001). Experiments were repeated two additional times. **C**. Bar graph presenting the mean (±SD) percentage of *RAD54B*-proficient (black) and *RAD54B*-deficient (grey) cells labeled with cleaved Caspase-3 following treatment with DMSO, Staurosporine (positive control), BMN673 or Olaparib. Student's *t*-tests reveal statistically significant increases in the percentage of *RAD54B*-deficient cells labeled with cleaved Caspase-3 following BMN673 and Olaparib treatments relative to corresponding *RAD54B*-proficient controls. (ns, not significant; *, *P*-value <0.05). Experiments were repeated two additional times.

Several research groups have shown that PARP inhibition induces DNA single strand breaks that are converted into DNA DSBs within HRR compromised cells to induce cell death [39, 40], while others have shown excessive DSBs can induce apoptosis [41]. To determine whether DSBs and apoptosis contribute to the PDs described above, we employed semi-quantitative, immunofluorescent microscopy [24] and quantified the global abundance of key indicators of DSBs (γ-H2AX [42]) and apoptosis (cleaved Caspase-3 [43]). We first established our ability to detect changes in γ-H2AX signal intensities (Figure 4A) using ionizing radiation (IR) as a positive control, and as expected, similar increases in γ-H2AX signal intensities occurred within both cell lines (Figure 4B). Interestingly however, although increases in the mean γ-H2AX signal intensities occurred in both lines following BMN673 and Olaparib treatments, the mean γ-H2AX signal intensities were consistently higher within the *RAD54B*-deficient cells (Figure 4B) suggesting increases in DNA DSBs occur preferentially within *RAD54B*-deficient cells. In fact, Student's *t*-tests revealed significant increases in mean γ-H2AX signal intensities within the *RAD54B*-deficient cells treated with either BMN673 (1.2-fold) or Olaparib (1.3-fold) relative to controls (Table S8).

Having established that BMN673 and Olaparib treatments preferentially induce increases in a surrogate marker for DNA DSBs in *RAD54B*-deficient cells, we now wished to determine whether it was associated with increases in apoptosis, as identified by cleaved Caspase-3 signal intensity. Accordingly, quantitative microscopy was performed and the percentage of cells labeled with cleaved Caspase-3 was determined, with Staurosporine serving as the positive control. As shown in Figure 4C, there is a small albeit statistically significant increase in the proportion of *RAD54B*-deficient cells labeled with cleaved Caspase-3 following BMN673 (1.4-fold) and Olaparib (3.0-fold) treatments relative to controls (Table S9). Collectively, these data show there is an increase in both DNA DSBs and apoptosis following PARP inhibition in *RAD54B*-deficient cells, that likely accounts for the decrease in cells numbers remaining following treatments.

### BMN673 and LCS-1 synergize to enhance *RAD54B*-deficient cell killing

**Figure 5:**
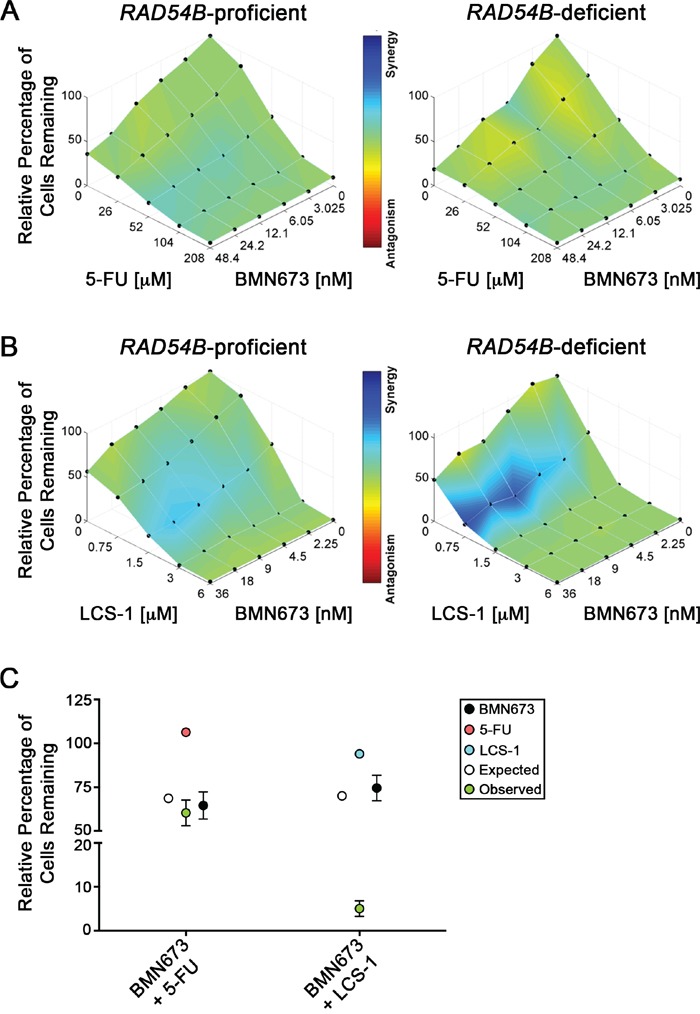
BMN673 synergizes with LCS-1 for enhanced killing of CRC cells Combenefit mapped surface output for the drug combinations involving BMN673 with **A**. 5-FU, or **B**. LCS-1, in *RAD54B*-proficient (left) and *RAD54B*-deficient (right) cells. The concentrations of each drug are plotted along the horizontal axes, while the percentage of cells remaining relative to DMSO-treated controls is plotted on the vertical axis. A heat map is used to represent the level of synergy (blue color) at each concentration. All experiments were conducted at least three times. **C**. Graphical depiction of the mean normalized percentage of *RAD54B*-deficient cells relative to DMSO (±SD), following single agent treatment with BMN673 (black circles) and either 5-FU (orange circles) or LCS-1 (blue circles), as well as the expected combined value calculated using a multiplicative model (white circles). Green circles identify observed values following combinatorial treatments with either BMN673 plus 5-FU, or BMN673 plus LCS-1. Note that the observed value for BMN673 plus LCS-1 indicates synergistic killing.

Recognizing the limited therapeutic potential of targeting PARP1 alone in *RAD54B*-deficient cells, we wished to identify drug combinations that would exacerbate the SL effect observed with BMN673. We hypothesized that combinations involving inhibitors of known SL interactors, like *SOD1* [24], would produce synergistic killing within *RAD54B*-deficient cells. Accordingly, we performed both single and dual agent dose response experiments involving BMN673 and LCS-1, a recently identified SOD1 inhibitor [44]. As a control, we included 5-FU, a classical frontline drug frequently employed in combinatorial approaches in CRC [45] that does not induce SL killing in *RAD54B*-deficient cells. Based on Combenefit analyses, the BMN673 plus 5-FU combination showed no synergy and was merely additive (Figure 5A). In stark contrast however, the BMN673 plus LCS-1 combination induced synergistic killing (Figure 5B) that was readily apparent at concentrations ranging from 2.25-36.0 nM (BMN673) and 0.75 μM (LCS-1).

Next, we performed modified colony formation assays (mCFAs) to extend the above findings and confirm the simultaneous administration of BMN673 and LCS-1 induces synergistic killing within the *RAD54B*-deficient cells. As shown in Figure 5C, the combination involving BMN673 plus 5-FU induced growth defects within *RAD54B*-deficient cells (60.3% confluent relative to DMSO treatment), but to approximately the extent predicted using a multiplicative model (68.7%). This represents a relatively small ∼12.2% difference between expected and observed values (Table S10), and supports the Combenefit findings above, suggesting an additive interaction. Interestingly however, the combination involving BMN673 and LCS-1 produced a large decrease in relative cell confluency beyond that predicted using the multiplicative model (Figure 5C). More specifically, the predicted (70.0%) and observed (5.0%) confluency for *RAD54B*-deficient cells represents a 92.8% difference (Table S11) and strongly indicates the BMN673 and LCS-1 combination is synergistic within *RAD54B*-deficient cells.

## DISCUSSION

To expand the number of drug targets of *RAD54B* and simultaneously assess the broad-spectrum applicability of PARP1 as a candidate drug target, we evaluated the ability of PARP1 silencing and inhibition to induce SL killing in *RAD54B*-deficient CRC cells. Using an isogenic *RAD54B* model, we show that PARP1 silencing preferentially reduces the number of *RAD54B*-deficient cells relative to controls. We further show that two PARP inhibitors, BMN673 and Olaparib, can functionally substitute for *PARP1* silencing by inducing a decrease in *RAD54B*-deficient cells relative to controls in both short (<4 days) and longer-term assays (<7 days). We further show that BMN673 and Olaparib treatments induce PDs in *RAD54B*-deficient cells that are accompanied by increases in γ-H2AX and cleaved Caspase-3, which are suggestive of increases in DNA DSBs and apoptosis, respectively. Collectively, these data confirm that *RAD54B* and *PARP1* are SL. Finally, to enhance the potential therapeutic utility and effect of PARP inhibitors, we explored combinatorial approaches involving BMN673 and either 5-FU or LCS-1. Here, we show that combining LCS-1, but not 5-FU, with BMN673 induced synergistic killing within *RAD54B*-deficient cells. Thus, this study identifies and validates PARP1 as a candidate drug target in *RAD54B*-deficient CRC cells, and identifies BMN673 and Olaparib as novel candidate chemotherapeutic agents in a *RAD54B*-specific CRC context. The results also support further preclinical studies evaluating the efficacy of combinatorial approaches involving BMN673 and LCS-1.

Since the original description of a SL interaction between *PARP* and *BRCA1/2* in 2005 [39, 40], PARP inhibitors have garnered substantial attention as novel compounds for precision medicine based approaches in the fight against cancer. In 2014, Olaparib was approved as a mono-therapy for maintenance in women with *BRCA1/2* mutant, platinum-responsive, high-grade serous ovarian cancer [46]. Moreover, since the original discovery significant research efforts have focused on expanding the number of chemogenetic interactions involving Olaparib and other PARP inhibitors. In 2014 for example, Bajrami *et al* [27] performed a genome-wide, shRNA drop-out screen to identify novel chemogenetic interactors of Olaparib. Although this screen confirmed several established *PARP1* interactors (*BRCA1, RAD51*, and *FANCC*) and identified many novel interactions, it did not confirm several established interactors (e.g. *BRCA2* and *DSS1*) nor did it identify *RAD54B* as a SL interactor. The authors suggested that they were unable to confirm the established interactions due to inadequate silencing, which may also account for why *RAD54B* was not previously identified. Independent of this work, many research laboratories have sought to expand the number of chemogenetic interactors of PARP inhibitors. Influenced heavily by the initial observations involving *BRCA1/* 2, a number of studies focused on HRR (or DDR) genes as putative interactors. Indeed, many of the identified interactors encode functions within HRR (or DDR) including *RAD54L* [27]*, NBS* [27, 30]*, RAD51* [27, 30]*, RAD51C* [27, 31]*, RAD51D* [27, 32]*, MRE11A* [33]*, XRCC1* [34]*, XRCC2* [27]*, ATR* [30]*, ATM* [30]*, DSS1* [30]*, FANCC* [30] and *CHEK2* [30], and were identified through a combination of high-throughput screens [27, 28] and direct tests [30–33]. Thus, through direct tests, we have expanded this list to include *RAD54B*.

Although the clinical applicability of PARP inhibitors is still in its infancy, mechanisms of PARP inhibitor resistance have been identified. For instance, Olaparib resistance was conferred in *BRCA2*-deficient cancers harboring frame-shift mutations through subsequent mutations that restored the *BRCA2* reading frame to effectively/partially rescue the HRR defect [47]. Thus, a major avenue of scientific inquiry now focuses on identifying and predicting the mechanisms of drug resistance in the hopes of identifying additional drug targets that will prevent the resistance mechanism. A major limitation of many current cancer therapies is the risk of developing resistant disease following multiple courses with a particular drug resulting from the selection of a pre-existing resistant clone or the development of a resistant clone within the tumor. It has been suggested that disease recurrence and resistance may be minimized or avoided through the use of combinatorial chemotherapeutic approaches rather than sequential application of single agents [48, 49]. Conceptually, combinatorial approaches may target more cells within the tumor, thus reducing the potential for selection and/or development of resistant clones. In oncogenic addiction for example, drug resistance can occur as a result of redundant biological pathways or the compensatory activity of a secondary pathway that circumnavigates the inhibitory activity of a drug to ultimately activate the downstream components of the oncogenic pathway. Consequently, combinatorial approaches that simultaneously target multiple biological pathways may negate or minimize this possibility and/or induce toxicity in more cells to prevent resistance from developing.

Combinatorial drug strategies beyond preventing drug resistance may also improve the overall treatment efficacy relative to single agents by producing more extensive and robust killing of cancer cells within a tumor. For example, identifying synergistic drug combinations that produce therapeutic effects that are greater than the sum of the two individual agents alone would be highly beneficial. In the current study, we determined BMN673 in combination with LCS-1 induces synergistic killing within *RAD54B*-deficient cells, whereas BMN673 with 5-FU was only additive. Thus, these findings suggest that generalized DNA damage may be insufficient to synergize with PARP1 inhibition, but rather, synergy may depend on the particular mechanism of DNA damage induction and the particular proteins or factors involved. Importantly, these results demonstrate how chemotherapeutic agents targeting different SL pathways may be strategically combined to enhance the overall SL effect. The same theoretical strategy could be applied to other SL targets and in CRC and other cancer types to more efficiently target and kill cancer cells.

As we approach the era of precision medicine, identifying SL interactors and synergistic drug combinations may represent superior therapeutic strategies to traditional approaches that tend to indiscriminately target all replicating cells, including cancer and normal. Thus, a major goal of the current study was to determine whether *PARP1* was a SL interactor of an additional gene involved in HRR. Indeed, we showed that *PARP1* is SL with *RAD54B*, and further determined that BMN673 and Olaparib are capable of exploiting a *RAD54B*-deficiency in a CRC context. We further evaluated the efficacy of BMN673 in combination with other chemotherapeutic agents, and identified a drug combination (BMN673 plus LCS-1) that induces synergistic killing in 2D cell culture models. Thus, these initial studies may serve as the underpinning for subsequent preclinical work aimed at evaluating the efficacy of these agents within more complex systems, such as animal models. Additionally, these findings serve as a proof of concept in support of combinatorial chemotherapies involving multiple SL targets for a single gene altered in cancer. Thus, expanding SL networks in general and extending these strategies to include additional cancer types may hold clinical potential within the broader context of anti-cancer therapies.

## MATERIALS AND METHODS

### Cell culture

HCT116 (*RAD54B*-proficient) cells were purchased from American Type Culture Collection. *RAD54B*-deficient HCT116 cells were generously provided by K. Miyagawa (Hiroshima University, Japan) and were generated by targeted integration and disruption of the *RAD54B* locus [11]. All HCT116 cells were grown in McCoy's 5A medium (HyClone) supplemented with 10% FBS, and RAD54B expression was confirmed by Western blot (Figure 1A). Cell lines were authenticated on the basis of recovery, viability, growth and morphology, and spectral karyotyping. All cells were grown in a 37°C humidified incubator containing 5% CO_2_.

### Gene silencing

Cells were transiently transfected with siRNA duplexes using RNAiMax (Invitrogen) as detailed elsewhere [19, 24]. ON-TARGETplus (Dharmacon) siRNA duplexes targeting *RAD54B*, *PARP1*, *GAPDH* and *PLK1* were employed as either individual duplexes or pools (four distinct duplexes targeting the gene of interest), as detailed previously [19, 24]. Gene silencing was confirmed by Western blot.

### Western blotting

Western blots were performed as detailed elsewhere [19] and blotted with RAD54B (1:1000; provided by Dr. K. Miyagawa), PARP1 (Abcam ab6079; 1:7500), and α-tubulin (Abcam ab7291; 1:20,000) antibodies. Semi-quantitative analyses were employed to evaluate silencing efficiencies using the Gel Analyzer Tool in ImageJ. All data were normalized to the corresponding loading control (α-tubulin) and are presented relative to the negative control (si*GAPDH*).

### Direct SL tests

High-content microscopy was used to evaluate the SL interaction as detailed elsewhere [19, 24]. Briefly, 4000 *RAD54B*-proficient or *RAD54B*-deficient HCT116 cells were seeded into 96-well optical plates. Cells were transfected in sextuplet (i.e. 6 wells per plate) with either individual or pooled siRNAs targeting *RAD54B*, *PARP1*, and controls (*GAPDH* and *PLK1*). *GAPDH* serves as a negative control [19], while *PLK1* is a positive control for cell death independent of any SL interaction [50] and also serves as a transfection efficiency indicator. Wells were supplemented with 100 μL of media 24 hour (h) post-transfection, and permitted to grow for an additional 3.5 days, following which cells were fixed (4% paraformaldehyde), and counterstained with Hoechst 33342 (300 ng/mL; Thermo Scientific). Images were acquired using a Cytation 3 (BioTek) equipped with a 10x objective (0.3 numerical aperture), a 16-bit gray scale charged couple device camera and Gen5 software. Nine central, non-overlapping images were acquired per well (i.e. condition), and the total number of cells remaining in each well was determined. All data were imported into Prism v6.0 (GraphPad), normalized to GAPDH silenced controls, and basic statistical analyses (e.g. mean, standard deviation, Student *t*-tests) were performed as described [24]. To address reproducibility all experiments were conducted a minimum of three times.

### Dose response curves

Standard dose response curves were generated using a 10-fold serial dilution of BMN673 (200 fM to 20 μM), Olaparib (2 pM to 200 μM), a 5-fold serial dilution of 5-FU (1.28 nM to 100 μM), or a 2-fold serial dilution of LCS-1 (46.9 nM to 12 μM). Briefly, 4000 *RAD54B*-proficient or -deficient cells were seeded into 96-well optical plates, permitted to attach and treated 24 h post-seeding with DMSO (vehicle control), BMN673, Olaparib, 5-FU, or LCS-1. Cells were permitted to grow for an additional 3.5 days, at which point they were fixed, counterstained (Hoechst), imaged, and analyzed as above. Imaging data (i.e. nuclear counts) were imported into Prism v5.0 (GraphPad) and normalized to DMSO control and an Effective Concentration 50 (EC_50_) value was determined. The EC_50_ values for BMN673 (9 nM), Olaparib (2.6 μM), 5-FU (52 nM) and LCS-1 (1.7 μM) in *RAD54B*-deficient cells were employed in all subsequent experiments. All experiments were conducted a minimum of three times.

### Real time cell analyses

Real-time cell analyses (RTCA) (i.e. proliferation curves) were performed in quadruplicate using an RTCA-dual plate (RTCA-DP; Acea Biosciences) instrument housed within a 37°C incubator. The RTCA-DP system employs microelectrodes at the bottom of each well to measure increases or decreases in electrical impedance, termed cell index that reflect increases or decreases in cell numbers, respectively. Briefly, 4000 cells were seeded into each well of an E-plate and growth was monitored every 15 min. DMSO, BMN673 or Olaparib were supplemented into the appropriate wells 24 h post seeding, and growth was monitored for ∼4 days. All data were imported into Prism, where Proliferation Defects (PD) were calculated for each line and condition using the following formula:

$$\text{PD}=\left\{1-\left\{\frac{{\text{Cell Index}}_{\text{Drug}}}{{\text{Cell Index}}_{\text{DMSO}}}\right\}\right\}\times 100$$

### Modified two dimensional colony forming assays

Modified colony forming assays (mCFAs) were performed utilizing a cell seeding density of 1000 cells/well. Briefly, cells were seeded and treated 24 h later in sextuplet with DMSO, BMN673, or Olaparib. Cells were permitted to grow for 7 days, following which they were fixed, counterstained (0.005% crystal violet solution; Sigma) and imaged. Image intensity thresholding was applied to generate a binary mask that was employed to quantify cell confluency. Next, a circle of a fixed diameter was applied to each well and the average pixel intensity was determined and presented relative to the corresponding control. Similar mCFAs were performed for combinatorial drug treatments, where the media were supplemented with DMSO, BMN673, LCS-1, 5-FU, or combinations of BMN673 plus 5-FU or BMN673 plus LCS-1. Drug concentrations and cell seeding densities were optimized to achieve appropriate growth within a quantifiable range (9 nM BMN673, 52 nM 5-FU and 187.5 nM LCS-1 at 2000 cells/well). To address reproducibility all experiments were conducted a minimum of three times.

### Quantitative imaging microscopy

The presence of DNA DSBs was evaluated using an established quantitative, indirect immunofluorescence (IIF) approach [51]. Briefly, 120,000 cells were seeded onto coverslips and permitted to attach. Cells were treated with media containing BMN673, Olaparib or DMSO 24 h post cell seeding and grown for an additional 24 h. Ionizing radiation (IR; 2 Grey) was used as a positive control for DNA DSBs using an RS 2000 X-ray Irradiator (RAD Source Technologies). Cells were fixed, permeabilized, immunofluorescently labeled with γ-H2AX (Abcam ab26350; 1:200) antibody, counterstained (DAPI) and imaged using identical exposure times as described [51]. The total γ-H2AX signal intensity was determined for each nucleus from a minimum of 175 nuclei/condition. Apoptosis was similarly assessed and quantified by calculating the percentage of cells labeled with a cleaved Caspase-3 antibody (Abcam ab13847; 1:200). Briefly, cells were seeded as above and treated with Staurosporine (1 μM; positive control), DMSO, BMN673 or Olaparib. Cells were fixed, permeabilized, immunofluorescently labeled and a minimum of 500 nuclei/condition were imaged and evaluated.

### Multi-agent dose response

To assess multi-drug combinations a similar protocol to the *Dose Response Curve* (above) was employed. Briefly, 4000 asynchronous cells were seeded into each well of a 96-well optical plate. After 24 h, media were supplemented with DMSO (vehicle control), BMN673, 5-FU, LCS-1, or a combination of BMN673 plus 5-FU, or BMN673 plus LCS-1. Five distinct concentrations were employed for BMN673 (2.25, 4.5, 9, 18, 36 nM), while four concentrations were employed for 5-FU (26, 52, 104, 208 nM) and LCS-1 (0.75, 1.5, 3, 6 μM). Cells were permitted to grow for 3.5 days at which point they were analyzed as described above. All data were normalized to DMSO treated controls, and imported into Combenefit software where the Loewe Additivity model was employed to identify synergistic, additive or antagonistic drug combinations.

### Multiplicative model

A multiplicative model was employed to determine whether the combined effects of two individual treatment conditions were greater than that predicted by the product of the individual treatments, and is:

A×B=E

where A is the relative percentage of cells remaining following condition A, B is the relative percentage of cells remaining following condition B and E is the expected product of the combined treatments. If the observed relative percentage of cells remaining is less than that predicted by the multiplicative model (i.e. fewer cells remaining within the combined condition than predicted by the model), the response of the combined treatment conditions is synergistic.

## SUPPLEMENTARY FIGURE AND TABLES


