# Supplementary material for: The synthetic lethal killing of RAD54B-deficient colorectal cancer cells by PARP1 inhibition is enhanced with SOD1 inhibition
Source: Oncotarget. 2016 Nov 26;7(52):87417–30. doi: 10.18632/oncotarget.13654 (PMC5349998; doi:10.18632/oncotarget.13654)
Supplement: Supplementary file 1 [file oncotarget-07-87417-s001.pdf]

The synthetic lethal killing of *RAD54B*-deficient colorectal cancer cells by PARP1 inhibition is enhanced with SOD1 inhibition

SUPPLEMENTARY FIGURE AND TABLES

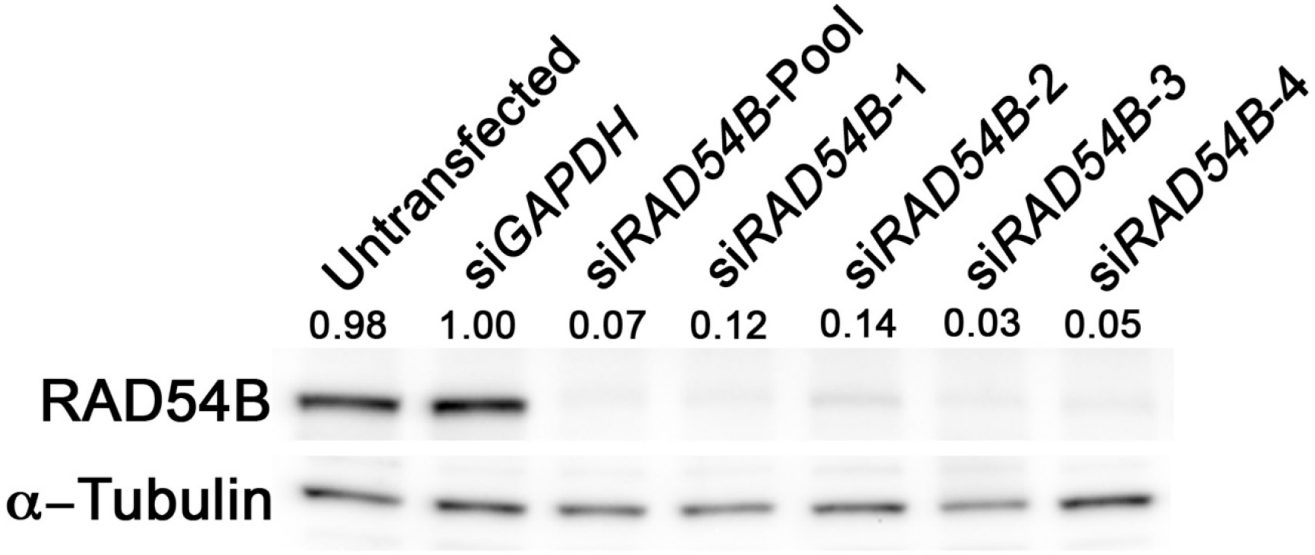

Supplementary Figure S1: Efficiency of si*RAD54B*-based Silencing in HCT116 (*RAD54B*-Proficient) Cells. Western blot showing diminished RAD54B expression following silencing relative to controls (Untransfected and si*GAPDH*); α-Tubulin serves as the loading control. *RAD54B* expression levels are indicated above each lane and are presented relative to the si*GAPDH* control. Experiments were repeated at least two additional times.

Supplementary Table S1: Student's *t*-tests identifying statistical differences in relative percentage of cells remaining following PARP1 silencing between *RAD54B*-proficient and *RAD54B*-deficient cells

siRNA treatment	n[A]	Mean cell number ± SD[B]		Mean normalized relative percentage[C]		*P*-value
		RAD54B-proficient	*RAD54B*-deficient	*RAD54B*-proficient	*RAD54B*-deficient	
si*GAPDH*	6	14903 ± 303.1	15870 ± 332.7	100 ± 2.034	100 ± 2.097	> 0.9999
si*PARP1*-P	6	14355 ± 296.9	14571 ± 523.0	96.32 ± 1.992	91.81 ± 3.296	0.0167
si*PARP1*-1	6	13568 ± 267.4	13884 ± 363.8	91.04 ± 1.794	87.48 ± 2.292	0.0135
si*PARP1*-2	6	14504 ± 551.7	14462 ± 335.6	97.32 ± 3.702	91.13 ± 2.114	0.0052
si*PLK1*	6	1074 ± 405.8	925 ± 579.9	7.206 ± 2.723	5.831 ± 3.654	0.4766

[A]n; number of wells analyzed.
[B]SD; standard deviation.
[C]All values are normalized to si*GAPDH* control for respective cell line.

Supplementary Table S2: Dual siRNA-based synthetic lethal testing in HCT116 cells

siRNA treatment	n[A]	Mean cell number ± SD[B]	Normalized relative percentage[C]	Expected percentage[D]	Percent difference[E]
si*GAPDH*	6	15356 ± 721.4	100 ± 4.698	N/A	N/A
si*RAD54B*	6	14130 ± 914.3	92.02 ± 5.955	N/A	N/A
si*PARP1*-P	6	13006 ± 967.0	84.70 ± 6.297	N/A	N/A
si*PARP1*-1	6	12791 ± 746.7	83.30 ± 4.863	N/A	N/A
si*PARP1*-2	6	12693 ± 354.1	82.66 ± 2.306	N/A	N/A
si*RAD54B* + si*PARP1*-P	6	8908 ± 1120	58.01 ± 7.293	77.938	26
si*RAD54B* + *siPARP1*-1	6	9515 ± 892.2	61.97 ± 5.811	76.654	19
si*RAD54B* + si*PARP1*-2	5	10220 ± 1440	66.56 ± 9.377	76.067	18
si*PLK1*	6	859 ± 45.11	5.596 ± 0.2938	N/A	N/A

[A]n; number of wells analyzed.
[B]SD; standard deviation.
[C]All values are normalized to si*GAPDH* control for respective cell line.
[D]Calculated by multiplying the normalized percentage of the two individual siRNAs.
[E]Calculated as 1 – (Normalized relative percentage/expected percentage) × 100 (N/A; not applicable).

Supplementary Table S3: Student's *t*-tests reveal statistical differences of relative percentage of cells remaining following BMN673 treatment within *RAD54B*-deficient cells compared to controls

Drug Treatment	n[A]	Mean cell number ± SD[B]		Mean normalized relative percentage[C] ± SD		*P*-value
		***RAD54B*-proficient**	***RAD54B*-deficient**	***RAD54B*-proficient**	***RAD54B*-deficient**	
DMSO	6	12791 ± 759.5	11743 ± 859.9	100 ± 5.938	100 ± 7.322	> 0.9999
2 nM BMN673	6	12509 ± 409.1	10306 ± 601.5	97.79 ± 3.198	87.76 ± 5.122	0.0022
20 nM BMN673	6	6911 ± 701.1	4933 ± 230.5	54.03 ± 5.481	42.01 ± 1.963	0.0005

[A]n; number of wells analyzed.
[B]SD; standard deviation.
[C]All values are normalized to vehicle control (DMSO) for respective cell line.

Supplementary Table S4: Student's *t*-tests identify statistical differences of relative percentage of cells remaining following olaparib treatment within *RAD54B*-deficient cells compared to controls

Drug Treatment	n[A]	Mean cell number ± SD[B]		Mean normalized relative percentage[C] ± SD		*P*-value
		***RAD54B*-proficient**	***RAD54B*-deficient**	***RAD54B*-proficient**	***RAD54B*-deficient**	
DMSO	6	14567± 506.5	15330 ± 525.4	100 ± 3.477	100 ± 3.427	> 0.9999
0.2 µM Olaparib	6	13032 ± 373.8	12982 ± 506.5	89.46 ± 2.566	84.68 ± 3.304	0.0386
2.0 µM Olaparib	6	10202 ± 386.8	9293 ± 449.4	70.03 ± 2.655	60.62 ± 2.931	0.0016

[A]n; number of wells analyzed.
[B]SD; standard deviation.
[C]All values are normalized to vehicle control (DMSO) for respective cell line.

Supplementary Table S5: Student's *t*-tests reveal statistical differences of relative percentage of cell confluency following BMN673 treatment within *RAD54B*-deficient cells compared to controls in modified 2D colony forming assays

Drug Treatment	n[A]	Mean normalized relative percentage[B] ± SD[C]		*P*-value
		RAD54B-proficient	*RAD54B*-deficient	
DMSO	6	100 ± 5.47	100 ± 5.81	> 0.9999
BMN673	6	36.76 ± 10.91	19.48 ± 2.09	0.0034

[A]n; number of wells analyzed.
[B] All values are normalized to vehicle control (DMSO) for respective cell line.
[C]SD; standard deviation.

Supplementary Table S6: Student's *t*-tests identify statistical differences of relative percentage of cell confluency following olaparib treatment within *RAD54B*-deficient cells compared to controls in modified 2D colony forming assays

Drug Treatment	n[A]	Mean normalized relative percentage[B] ± SD[C]		*P*-value
		RAD54B-proficient	*RAD54B*-deficient	
DMSO	6	100 ± 2.28	100 ± 3.95	> 0.9999
Olaparib	6	42.86 ± 15.04	15.73 ± 4.89	0.0018

[A]n; number of wells analyzed.

[B] All values are normalized to vehicle control (DMSO) for respective cell line.

[C]SD; standard deviation.

Supplementary Table S7: Proliferation defect (PD[A]) values calculated from RTCA proliferation curves

Inhibitor	*RAD54B*-proficient PD ± SD[C]	*RAD54B*-deficient PD ± SD[C]	Fold Increase[B]
BMN673	0.55 ± 0.05	51.80 ± 5.35	94.18
Olaparib	1.71 ± 0.18	17.83 ± 2.19	10.43

[A]PD; Proliferation defect.
[B]Fold increase; *RAD54B*-deficient PD/*RAD54B*-proficient PD.
[C]SD; standard deviation.

Supplementary Table S8: Student's *t*-tests identifying statistical differences of total γ-H2AX signal intensity following BMN673 and olaparib treatment

Treatment	n[A]	Mean total γ-H2AX signal intensity ± SD[B] (×10^6 A.U.)		*P*-value
		***RAD54B*-proficient**	***RAD54B*-deficient**	
IR	175	15.8 ±7.05	15.6 ± 7.59	0.8202
DMSO	175	6.23 ± 3.06	5.83 ± 2.7	0.1928
BMN673	175	7.52 ± 4.94	9.19 ± 6.23	0.0058
Olaparib	175	7.99 ± 4.40	10.6 ± 6.88	<0.0001

[A]n; number of cells evaluated.
[B]SD; standard deviation.

Supplementary Table S9: Student's *t*-tests identifying statistical differences of percent of cells with activated cleaved Caspase-3 signal within *RAD54B*-deficient cells following BMN673 and olaparib treatment

Drug Treatment	N[A]	Percentage of cells with activated cleaved Caspase-3 ± SD[B]		*P*-value
		RAD54B-proficient	*RAD54B*-deficient	
Staurosporine	2	2.66 ± 0.28	2.35 ± 0.19	0.3285
DMSO	2	0.05 ± 0.07	0.04 ± 0.05	0.8531
BMN673	2	0.50 ± 0.01	0.69 ± 0.03	0.0132
Olaparib	2	0.23 ± 0.08	0.70 ± 0.01	0.0125

[A]N; number of experimental replicates.
[B]SD; standard deviation.

Supplementary Table S10: BMN673 and 5-FU combinatorial drug treatments in *RAD54B*-deficient cells

Drug Treatment	n[A]	Mean normalized relative percentage[B] ± SD[C]	Expected percentage[D]	Percent difference[E]
DMSO	3	100 ± 3.69	N/A	N/A
BMN673	3	64.57 ± 7.73	N/A	N/A
5-FU	3	106.33 ± 2.12	N/A	N/A
BMN673 + 5-FU	3	60.31 ± 7.34	68.65	12.16

[A]n; number of wells analyzed.
[B]All values are normalized to vehicle control (DMSO).
[C]SD; standard deviation.
[D]Calculated by multiplying the normalized percentage of the two individual drugs.
[E]Calculated as 1 – (Normalized relative percentage/expected percentage) × 100 (N/A; not applicable).

Supplementary Table S11: BMN673 and LCS-1 combinatorial drug treatments in *RAD54B*-deficient cells

Drug Treatment	n[A]	Mean normalized relative percentage[B] ± SD[C]	Expected percentage[D]	Percent difference[E]
DMSO	3	100 ± 0.27	N/A	N/A
BMN673	3	74.50 ± 7.31	N/A	N/A
LCS-1	3	93.98 ± 1.75	N/A	N/A
BMN673 + LCS-1	3	5.034 ± 1.77	70.02	92.81

[A]n; number of wells analyzed.
[B]All values are normalized to vehicle control (DMSO).
[C]SD; standard deviation.
[D]Calculated by multiplying the normalized percentage of the two individual drugs.
[E]Calculated as 1 – (Normalized relative percentage/expected percentage) × 100 (N/A; not applicable).
